# Scattered and transmitted light as surrogates for activated carbon residual in advanced wastewater treatment processes: Investigating the influence of particle size

**DOI:** 10.1016/j.wroa.2024.100222

**Published:** 2024-04-09

**Authors:** Franziska Kirchen, Thomas Fundneider, Louis Gimmel, Michael Thomann, Michael Pulfer, Susanne Lackner

**Affiliations:** a Department of Civil and Environmental Engineering Sciences, Institute IWAR, Chair of Water and Environmental Biotechnology, Technical University of Darmstadt, Germany; b Mecana AG, Industriestrasse 39, 8864 Reichenburg, Switzerland; c Institute for Ecopreneurship, School of Life Sciences FHNW, Hofackerstraße 30, 4132 Muttez, Switzerland

**Keywords:** Adsorption, Pile cloth media filtration, Superfine powdered activated carbon, Filtration, Tertiary wastewater treatment, Flocculation

## Abstract

The use of powdered activated carbon (PAC) is a common process in advanced wastewater treatment to remove micropollutants. Retention and separation of PAC is essential as PAC loaded with micropollutants should not be released into the environment. Determining the activated carbon (AC) residual in the effluent poses a challenge, as there is currently no on-line measurement method. In this study, the correlation between turbidity, measured by scattered light, and absorption at wavelength of 550 nm (Absorption_550 nm_), measured by transmitted light, was investigated in relation to the AC residue. Linear correlations for turbidity (R^2^ = 0.95) and Absorption_550 nm_ (R^2^ = 1.00) to AC concentrations were observed in both laboratory and full-scale experiments in a pilot plant where superfine PAC was added prior to Pile Cloth Media Filtration (PCMF). Decreasing the particle size (d_50_) while maintaining the same AC concentration leads to increased turbidity: Therefore, a fourfold reduction in d_50_ results in a 2- to 3-fold increase in turbidity, whereas a 30-fold reduction in d_50_ leads to a 6-to 8-fold increase. Furthermore, the original wastewater turbidity led to a parallel shift in the linear correlation between turbidity and AC. Coagulant doses of up to 400 mg Me^3+^/g AC resulted in a 50% reduction in turbidity. However, higher concentrations from 400 to 1,000 mg Me^3+^/g AC resulted in increased turbidity with only a 30% reduction compared to the initial turbidity. The study also highlights the significance of AC particle size in optical measurements, impacting result accuracy.

## Highlights

• Turbidity showed a linear correlation with activated carbon concentration.
• The smaller the activated carbon size, the greater the turbidity.
• The original wastewater turbidity led to a parallel shift in the linear correlation.
• Coagulants change the turbidity at same activated carbon concentration.

## Graphical abstract

**Figure:**
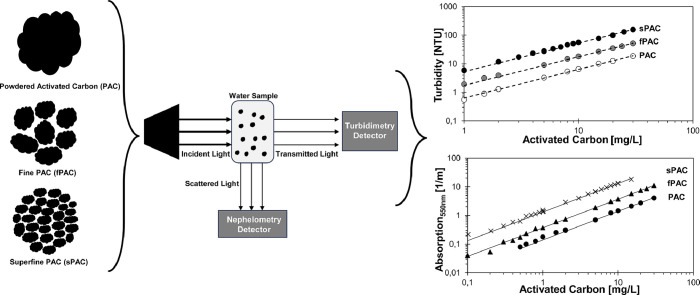

## 1 Introduction

Micropollutants such as pharmaceuticals, herbicides and pesticides are detected ubiquitously in the environment (Kim and Zoh 2016). These substances have a major negative impact on the flora and fauna of the environment, like toxicity to organs of mammals (Sathishkumar et al., 2021), endocrine toxicity to fish (Sumpter 2008) and much more. The effluents of municipal wastewater treatment plants (WWTP) as point sources contribute significantly to the pollution of water bodies, which makes an advanced treatment stage necessary, since conventional biological treatment does not remove micropollutants sufficiently (Tijani et al. 2013). For this purpose, oxidative processes with ozone or / and adsorptive processes with activated carbon are often used. Activated carbon (AC) can be used either in granular or powdered form.

Powdered activated carbon (PAC) is a finely ground AC and must have at least 95% of the mass fraction of a particle size smaller than 150 µm (DIN EN 12903, 2009). PAC can be used either by dosing into the activated sludge reactor (typically in nitrification stage) or by dosing it into separate contact reactors with subsequent removal of the PAC by coagulation, sedimentation and/or filtration. The particle size has a significant influence on the adsorption kinetics and it has been shown that smaller AC particles lead to a significantly faster removal of organic substances. Bonvin et al. (2016) showed that superfine PAC (sPAC) (d_50_ = 1 µm) just needs about 10 min to reach the adsorption equilibrium for different micropollutants, while the precursor PAC (d_50_=17 – 37 µm) needs about 12 h. The use of sPAC can lead to advantages in advanced treatment steps by reducing contact time between wastewater and AC, resulting in decreased construction volumes and AC consumption (Bonvin et al. 2016; Fundneider et al. 2023).

When using AC in WWTP, the effluent should have an AC concentration < 1 mg/L or AC removal > 95% to prevent loaded AC from entering water bodies and potentially harming the environment. PAC loaded with micropollutants in the effluent of advanced WWTP can also account for up to one third of the total micropollutant concentration in the effluent (Krahnstöver and Wintgens 2017a).

For the detection of particulate matter in wastewater, the parameter total suspended solids (TSS) is commonly used. The limit of quantification for TSS is 2 mg/L and lower concentrations cannot be determined with sufficient accuracy (DIN 38409–2:1987–03, 1987). Furthermore, the TSS removal does not correlate fundamentally with the AC removal, and is therefore not suitable for the quantitative assessment of the AC concentration in WWTP effluents (Löwenberg et al. 2016). Although low TSS concentrations indicate low AC concentrations, high TSS concentrations do not exclude good AC removal (Löwenberg et al. 2016).

Other methods, such as thermogravimetry (TGA) and gradient-TOC, are used for quantitative AC analysis in wastewater, while grey-scale imaging is limited to qualitative measurements only (DWA, 2023; Krahnstöver et al. 2016). But these methods do not enable continuous AC concentration measurements. Another method of quantifying suspended solids is the turbidity. Depending on the turbidity measurement method used (transmitted or scattered light measurement), differences in turbidity of more than a factor of 2 can be observed in the same sample (Gippel 1995). In addition, it has been shown that the sensitivity of turbidity sensors is higher for finer particles than for coarse particles (Merten et al. 2014). Previous attempts to use turbidity sensors to qualitatively assess residual AC concentrations in PAC treatment effluents have been made (Bornemann et al. 2012; Klaus and Wittmer, 2021; Metzger 2010; Wunderlin 2017). However, these efforts only served as an early detection of high AC concentrations in the effluent, indicated by operational disturbances of the treatment steps, and did not enable continuous quantitative and qualitative determination of residual AC concentrations. To use the turbidity more effectively for plant operation, the aim of this study was to investigate the correlation between turbidity and absorption at a wavelength of 550 nm (Absorption_550 nm_) and the AC concentration, and how the particle size of the AC, the background matrix and the use of coagulants influence the turbidity.

## 2 Results and discussion

### 2.1 Correlation between turbidity and activated carbon concentration

**Fig. 1:**
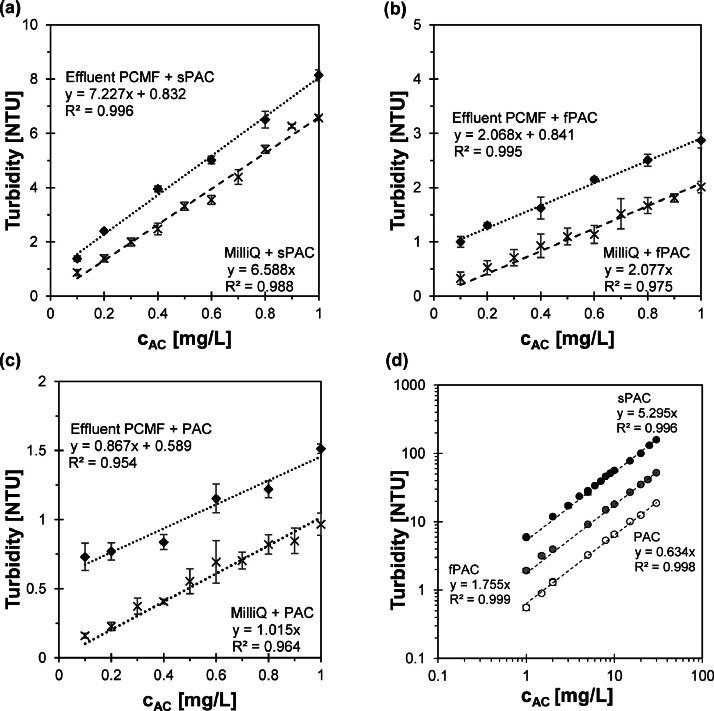
Correlation between turbidity and activated carbon (AC) with different particle sizes (PAC d_50_ = 31.2 µm; fPAC d_50_ = 7.6 µm; sPAC d_50_ = 1.0 µm) for concentrations up to 1 mg/L in MilliQ-Water and Effluent PCMF (Effluent of PCMF when no sPAC or coagulants were dosed with Turbidity_0_ = 0.73 NTU) (a, b, c) and in MilliQ-Water for concentrations from 1 to 30 mg/L (d).

The relationship between the turbidity measured with scattered light and the AC concentration for the three tested AC with different particle size distributions (PAC d_50_ = 31.2 µm; fine PAC (fPAC) d_50_ = 7.6 µm; sPAC (superfine PAC) d_50_ = 1.0 µm) is shown in Fig. 1(a)–(c). For all three AC types, turbidity and AC concentration exhibited a linear relationship for concentrations up to 1 mg/L in MilliQ-Water (R^2^ > 0.96). The slope of the relationship increased as the particle size of the tested AC became finer. Specifically, sPAC had the highest slope (6.59), followed by fPAC (2.08) and PAC (1.02). The effect of decreasing particle size leading to increasing turbidity at constant mass concentration has been demonstrated previously (Foster et al. 1992; Gippel 1988; Hernandez et al. 1991; Clifford et al. 1995). This relationship is due to the fact that as particle size decreases for a constant mass, the number of particles and the surface area to volume ratio increase. Even at higher concentrations of up to 30 mg/L, a linear correlation between double logarithmically plotted turbidity and AC concentration was observed for all three tested AC (Fig. 1(d)). The slopes of all three lines were up to 17% smaller compared to the slopes observed for AC concentrations up to 1 mg/L. Possible causes are that the increasing number of particles leads to increasing agglomeration due to more contact collisions of the particles or that there is an increasing effect of multiple scattering. SI Table 3 presents the grayscale measurements for various AC masses for the three tested AC. It is evident that as the particle size of the AC decreases, the intensity of greyness increases for a specific AC concentration. These results demonstrate that particle size significantly influences the grey-scale, causing the same mass of AC to appear very different with varying particle size distributions. Fig. 1(a)–(c) also present the correlation between turbidity and AC concentration, when AC was added in samples of the Pile Cloth media Filter (PCMF) effluent. The results show a parallel shift of the line for all three AC within the same range. These shifts can be attributed to the turbidity of the background matrix (Turbidity_0_). By subtracting Turbidity_0_ with 0.73 NTU from the y-axis intersection values of the three equations, the equations of the wastewater samples would overlap with the equations in MilliQ-Water. It is obvious that for higher Turbidity_0_ the values of the y-intercept will also be higher.

Turbidity measurements can vary significantly between different instruments. For instance, Anderson (2005) demonstrated that the turbidity of identical samples measured by different instruments could differ by a factor of 2 or more. In addition, Platz (2015) did turbidity measurements using the same type of activated carbon similar to this research (PAC, Carbopal AP) and found that a concentration of 20 mg/L in MilliQ-Water resulted in a turbidity value of 6 NTU, whereas Fig. 1(d) displayed a value of 13 NTU.

**Fig. 2:**
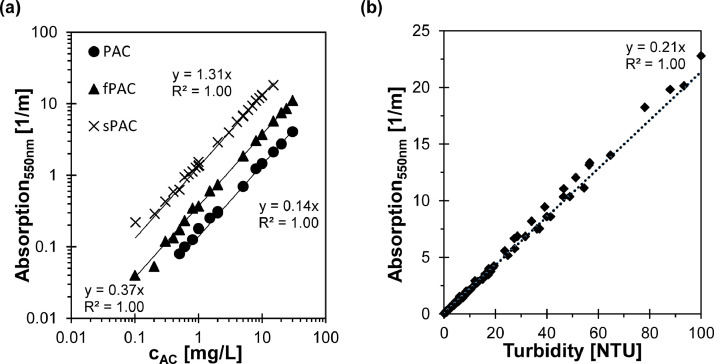
Correlation between Absorption at 550 nm and concentration of activated carbon (AC) with different particle sizes (PAC d_50_ = 31.2 µm; fPAC d_50_ = 7.6 µm; sPAC d_50_ = 1.0 µm) in MilliQ-Water (a) and correlation between Absorption at 550 nm and turbidity in MilliQ-Water (b).

The measurement of the Absorption_550nm_ for different AC concentrations is shown in Fig. 2(a). Again, a linear correlation for the AC concentration and the Absorption_550nm_ was visible and with finer particle size the Absorption_550nm_ for the same AC concentrations was higher. However, sPAC concentrations above 15 mg/L resulted in an extinction above 1.5 and a linear correlation was no longer evident. Similarly, no linear relationship between Absorption_550nm_ and AC concentration was observed for PAC concentrations below 0.50 mg/L.

A linear correlation was found between turbidity and Absorption_550nm_, with a relationship of Absorption_550nm_ [1/m] = 0.21 · turbidity [NTU] in MilliQ-Water, which is consistent with the findings of Gath and Rodenberg (2019), who also reported a value of 0.2.

### 2.2 Specific turbidity and particle size distribution

**Fig. 3:**
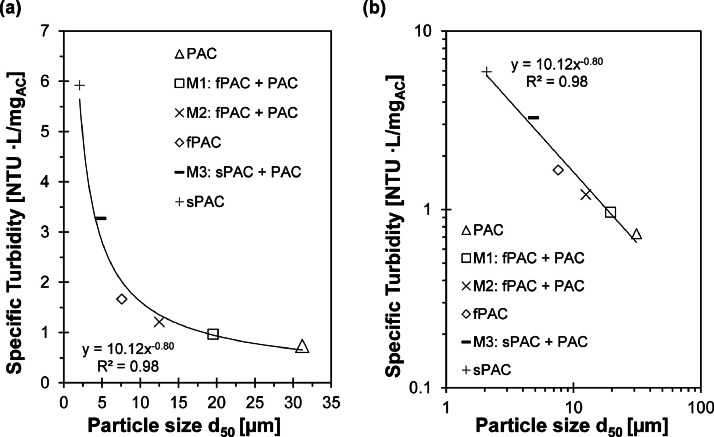
Correlation between specific turbidity and particle size (a) and double logarithmic representation of the correlation between specific turbidity and particle size (b) for three different powdered activated carbons with different particle sizes and powdered activated carbon mixtures (M1 (75% PAC + 25% fPAC), M2 (50% PAC + 50% fPAC), M3 (75% + 25% sPAC)).

The d_50_ values for the three AC types show a decrease from 2.1 to 1.0 µm for sPAC after sonication, while fPAC remained unchanged and PAC showed a small shift from 31.2 to 30.1 µm. This indicates that the primary particles of the sPAC tend to agglomerate, resulting in the formation of secondary particles (Decrey et al. 2020; Baalousha 2009; Dempsey 2006). And with the help of sonication, the secondary particles formed are disaggregated back to the initial primary particles. However, there is still uncertainty about the particle size distribution of the secondary particles after dosing sPAC into the wastewater and subsequent agglomeration. The agglomeration within the wastewater promotes effective separation, while at the same time there is no evidence that the adsorption capacity is reduced by the agglomeration (Bonvin et al. 2016; Decrey et al. 2020). Fig. 3 shows the specific turbidity (turbidity / AC concentration) in relation to the particle size. Particle size was determined for the AC without sonication, assuming that agglomeration of AC particles had occurred prior to the turbidity measurements. An almost linear increase in specific turbidity with decreasing particle size was observed between PAC, fPAC and the mixtures of fPAC and PAC. However, from a d_50_<6 µm, the non-linear influence of the particle size on the turbidity measurement became visible. In summary, the smaller the d_50_ of the AC, the higher its influence on the turbidity. The double logarithmic plot of Fig. 3(b) shows a linear relationship between specific turbidity and particle size. Foster et al. (1992) made similar observations for the specific turbidity of sediment particles with different particle sizes. Gippel (1988) found an exponential increase in specific turbidity with particle sizes up to 1.2 – 1.7 µm, while a particle size smaller than 1.2 µm led to an exponential decrease in specific turbidity, defining this as the critical particle size. The d_50_ of the AC used in this study, measured without sonication, was 2.1 µm, and no decrease in specific turbidity was observed. Therefore, it can be assumed that the samples did not reach the critical particle size, and additional reduction in particle size is expected to result in a decrease in specific turbidity.

Using turbidity as a surrogate for AC concentration, the exponential relationship indicates that turbidity is more sensitive when the particle size is closer to the critical particle size. For particle sizes larger than 20 µm, such as PAC used in advanced treatment steps, the difference in specific turbidity for increasing particle sizes was minimal, and a change in particle size does not significantly affect specific turbidity. However, this relationship is not universally valid for increasing particle sizes and is limited when particles become too large for measurement. The upper limit of this relationship still needs to be determined.

### 2.3 Influence of flocculation of powdered activated carbon on the turbidity

**Fig. 4:**
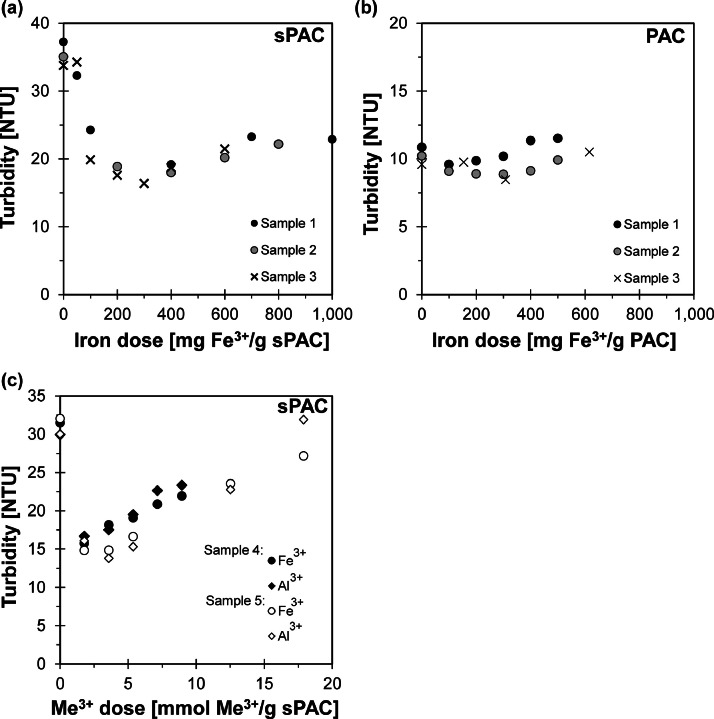
Turbidity and its behaviour during flocculation experiments with sPAC (10 mg/L) + FeCl_3_ (a) and PAC (10 mg/L) + FeCl_3_ (b) in three different wastewater samples. (c) shows the turbidity in flocculation experiments with sPAC (10 mg/L) and FeCl_3_ as well as AlCl_3_ (Turbidity_0_: Sample 1 = 2.77 NTU; Sample 2 = 2.85 NTU; Sample 3 = 2.26 NTU; Sample 4 = 1.59 NTU; Sample 5 = 3.68 NTU). All samples are from the PCMF influent without AC and coagulant dosing.

By using PAC in advanced wastewater treatment stages, the removal of the PAC is necessary and flocculation with metal salts is a common process. Typical dosages to flocculate PAC efficiently are between 100 and 400 mg Me^3+^/g PAC (Boehler et al. 2012; Tchobanoglous et al. 2014). Fig. 4 demonstrates the effect of flocculation of sPAC and PAC with FeCl_3_ on turbidity. Fig. 4(a) shows that the dosing of iron up to 400 mg Fe^3+^/g sPAC (5.37 mmol Fe^3+^/g sPAC) in three different wastewater samples with 10 mg/L sPAC led to a reduction in turbidity of about 50%. This reduction in turbidity, although the sPAC concentration remained the same, originated from the flocculation that produced larger particles which caused less turbidity. Therefore, this relation can also be used to determine the coagulant dosage required to achieve efficient flocculation of the sPAC, resulting in optimised floc separation by filtration. Krahnstöver and Wintgens (2017b) demonstrated that during flocculation of PAC with 1 mg Fe^3+^/L (Ironchloridsulfate), particle sizes < 10 µm decreased by about 80% after 15 min, while particle sizes of 10 – 100 µm increased by about 40% and also particles > 100 µm were formed. Above 400 mg Fe^3+^/g sPAC, increased turbidity caused by hydroxide formation was observed (Fig. 4 (a)). It is important to consider that the Fe^3+^/sPAC ratios found are wastewater-specific and depend on the soluble Reactive Phosphorus (sRP) concentration. Higher sRP concentrations require more coagulants.

In contrast to sPAC, no significant decrease in turbidity was observed at doses up to 400 mg Fe^3+^/g PAC in wastewater samples with a PAC concentration of 10 mg/L (Fig. 4(b)). Only doses above 400 mg Fe^3+^/g PAC resulted in an increase in turbidity of up to 15% compared to the sample without coagulant, which can be attributed to the formation of hydroxides. These results support the previously established correlation between specific turbidity and particle size. Once the particle size reaches approximately 20 µm, further changes in particle size do not have a substantial effect on specific turbidity. As the average particle size of the PAC was already 31.2 µm, the formation of larger particles by flocculation does not indicate a significant change in turbidity. In contrast, the d_50_ of the sPAC is 1 µm and any change in size due to flocculation will have a significant effect on the specific turbidity. Fig. 4(c) shows that flocculation of 10 mg/L sPAC in wastewater samples produces the same change in turbidity whether Fe^3+^ or Al^3+^ is used at the same concentrations.

### 2.4 Residual activated carbon concentration in the effluent of PCMF

**Fig. 5:**
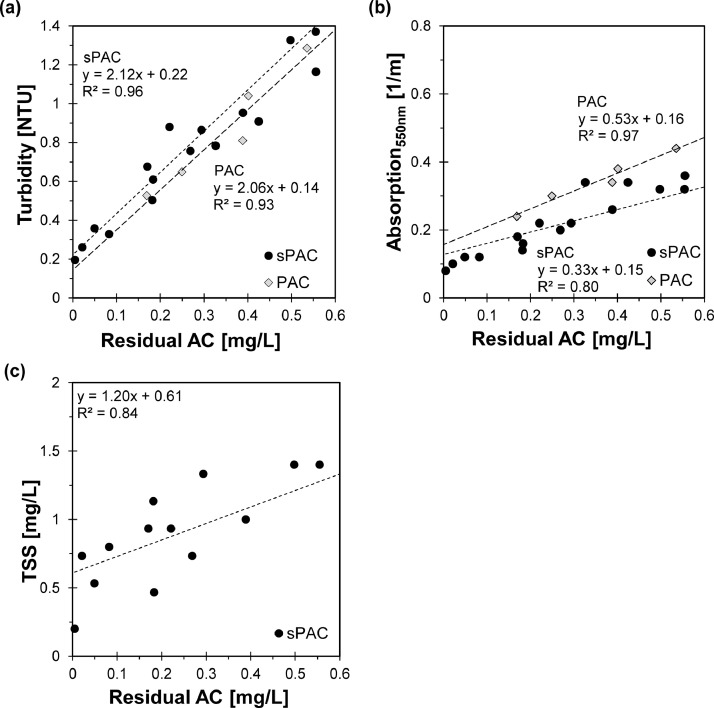
Correlation of the residual Activated Carbon (AC) concentration and turbidity (a), Absorption at 550 nm (b) and Total Suspended Solid (TSS) concentration (c) in the effluent of the PCMF when sPAC and PAC were dosed

The analysis of the residual AC concentration in the PCMF effluent was carried out for PCMF operation with sPAC and PAC. The correlation between turbidity and the residual AC is shown in Fig. 5(a). Again, there was a linear relationship between the turbidity and the AC concentration for sPAC (R^2^ = 0.96) and for PAC (R^2^ = 0.93). Because of the turbidity of the wastewater background matrix, the correlation line was shifted on the y-axis by about 0.14 NTU (PAC) respectively 0.22 NTU (sPAC). Despite the use of different FeCl_3_ concentrations ranging from 100 to 600 mg Fe^3+^/g AC in the PCMF influent, no deviation in the linear correlation was observed.

In contrast to the batch tests (Fig. 1), the turbidity of residual concentrations of sPAC and PAC showed a very similar slope of the line of 2.06 (PAC) and 2.12 (sPAC). This discrepancy can be explained by the fact that the mathematical image processing analysis of the activated carbon (MIPA^2^C) did not include particle size variations, resulting in different specific turbidities between sPAC and PAC. It can therefore be assumed that the concentrations of the residual sPAC concentrations must have been even smaller than the measured concentrations by the MIPA^2^C-method. Furthermore, the difference in the slopes of the correlation between turbidity and AC concentrations in MilliQ-Water (Fig. 1) and the effluent of the PCMF (Fig. 5) clearly indicates that the linear relationship established in MilliQ-Water was not suitable for determining AC in the PCMF effluent. This can be attributed to the fact that the particle distribution of AC has changed during the operation of the PCMF, due to the flocculation and separation processes carried out by the PCMF, in comparison to the original AC particle size distribution.

In addition to turbidity, the effluent samples when sPAC was dosed were also analysed for TSS and Absorption_550nm,_ while when PAC was dosed only Absorption_550nm_ was measured. For TSS (Fig. 5(b)), an increase in TSS concentration with an increase in the AC concentration occurred, but the results show a high variance and the results of the TSS concentration were < 2 mg/L and according to DIN 2:1987–03 (1987) under the limit of determination. For the Absorption_550nm_, a linear relationship to residual AC with R^2^ = 0.80 (sPAC) respectively R^2^ = 0.97 (PAC) were found. Again, a slight difference of 0.20 in the slope of the correlation lines between the two types of AC was observed, with the difference once again being significantly smaller than in MilliQ-Water. Other wave lengths like 436 nm, 525 nm or 620 nm did not show a sufficient correlation to residual AC concentrations.

## 3 Conclusion

When using AC in advanced wastewater treatment processes, there is the possibility of residual AC in the effluent. So far there is no method that can measure the AC concentration in the effluent continuously. This research dealt with the question whether the parameters turbidity and Absorption_550nm_ were suitable for determining the AC concentration. The following conclusions were reached:

• The turbidity (measured with scattererd light) and Absorption_550nm_ (measured with transmitted light) showed a linear correlation with the AC concentration, whereby the smaller the particle size of the AC, the greater the turbidity respectively Absorption_550nm_.
• A fourfold reduction in the d_50_ of AC led to a 2- to 3-fold increase in turbidity, while a 30-fold reduction in d_50_ of AC resulted in a 6- to 8-fold increase.
• The turbidity can indicate the concentration of AC, but it is more sensitive to changes in particle size when the particle size is about d_50_ < 6 µm. For larger particles than 20 µm, like PAC, changes in particle size have a minimal impact on turbidity.
• In optical AC measurements, like MIPA^2^C-method, AC particle size must be considered. Otherwise, the colour values determined cannot be accurately related to the corresponding mass of AC.
• In order to use turbidity as a surrogate for AC concentration, it must be considered that it is influenced by the wastewater matrix and coagulant use. The background matrix shifts the line on the y-axis by the amount of turbidity. The use of coagulants with sPAC reduced turbidity at concentrations between 300 and 500 g Me^3+^/g AC about 50% (compared to the initial turbidity), but higher coagulant concentrations increased the turbidity again, with only a 30% turbidity reduction (compared to the initial value). However, the correlation between turbidity and residual AC in the PCMF effluent was not influenced by coagulant use.

## 4 Material and methods

### 4.1 Activated carbon

The experiments were performed with lignite coal based AC (Donau Carbon, Carbopal AP) with three different particle sizes. More details on the AC used can be found in SI Table 1. The sPAC was produced by wet milling from the PAC using a ball mill (NETZSCH-Feinmahltechnik GmbH, Germany) with 0.7 – 0.8 mm grinding beads (ZrO_2_). The suspension of the sPAC had a concentration of 10% with a density of 1000 kg/m^3^. The particle size distributions of the three AC are shown in SI Fig. 1 and the method for analysing the particle size distribution is shown in Section 4.6. Table 1 illustrates the values of the particle diameter at 10% (d_10_), 50% (d_50_) and 90% (d_90_) of the cumulative distribution from the measurements with and without sonication. Also, mixtures of the three AC were prepared (M1 (75% PAC + 25% fPAC), M2 (50% PAC + 50% fPAC), M3 (75% + 25% sPAC)).

**Table 1:** Particle diameter at 10 % (d10), 50 % (d50) and 90 % (d90) of the cumulative distribution of the three used activated carbons from measurements with and without sonication.

Activated carbon		Measurement without sonication			Measurement with sonication		
		d_10_	d_50_	d_90_	d_10_	d_50_	d_90_
		[µm]	[µm]	[µm]	[µm]	[µm]	[µm]
PAC	Powdered activated carbon	4.4	31.2	88.4	4.6	30.1	85.8
fPAC	Fine powdered activated carbon	2.2	7.6	18.3	2.0	7.6	18.4
sPAC	Superfine powdered activated carbon	1.1	2.1	3.8	0.4	1.0	2.2

### 4.2 Laboratory experiments

Suspensions with the three different AC products were prepared with MilliQ-Water for the Laboratory experiments. While the sPAC was already in a liquid suspension after the wet milling as described in Section 4.1. (10%, ρ ∼ 1.000 kg/m³), the fPAC and PAC were measured with a precision balance (CP 124S Sartorius AG) and diluted with MilliQ-Water. Different volumes of the AC suspensions were added into MilliQ-Water as well into effluent of the PCMF (when no sPAC and coagulant was dosed in the pilot plant) to reach concentrations of 0.1 to 30 mg AC/L. After manual mixing, the turbidity of the sample was measured three times for every sample.

### 4.3 Flocculation experiments

For the flocculation tests, an AC concentration of 10 mg/L for PAC and sPAC was investigated. Different coagulant concentrations between 0 and 1000 mg Me^3+^/g AC (0 – 18 mmol Me^3+^/g AC) respectively 0 – 10 mg Me^3+^/L were used, which covered the typical range for PAC coagulation in WWTP application of 100 to 400 mg Me^3+^/g PAC (Boehler et al. 2012; Tchobanoglous et al. 2014). Both, iron(III)chloride (FeCl_3_, 13.8% Fe^3+^, Aqua-Technik GmbH) and aluminium(III)chloride (AlCl_3_, 9% Al^3+^, Aqua-Technik GmbH) were used. The flocculation tests were carried out by using a Jar Test (Flocculator SW6, Stuart) following the methodology outlined in Fundneider et al. (2020). In all tests, a first phase with high turbulence (250 rpm, 3 min) and a second phase with low turbulence (100 rpm, 15 min) were realized. Turbidity measurements were taken immediately after the 15-minute period to ensure that no sedimentation or additional flocculation had occurred.

### 4.4 Advanced wastewater treatment design

**Fig. 6:**
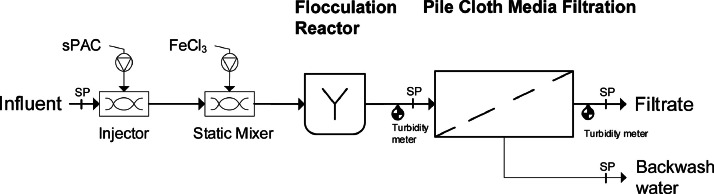
Advanced wastewater treatment setup with injector and static mixer for dosing superfine Powdered Activated Carbon (sPAC) and iron(III)chloride (FeCl_3_), followed by a flocculation reactor (V = 0.55 m^3^) and Pile Cloth Media Filter (PCMF). Sampling points (SP) at the influent of the pilot plant, at the influent and at the effluent of the PCMF.

Samples for the analysis of the residual AC concentration were taken from a pilot plant. The pilot plant treated the effluent from the secondary clarifier of a municipal wastewater treatment plant (WWTP) in southern Germany, which had a conventional activated sludge treatment. The flow chart is shown in Fig. 6. sPAC (5 – 15 mg/L) and FeCl_3_ (13.8% Fe^3+^, Aqua-Technik GmbH, 0 – 1900 mg Fe^3+^/g sPAC) were added into the effluent of the WWTP using an injector and a static mixer (Fluitec mixing + reaction solutions AG, Switzerland). The dosing of AC and FeCl_3_ were done by a peristaltic pump (Watson-Marlow AG, United Kingdom). After the static mixer, the wastewater had a hydraulic retention time of 0.5 – 2 min in a flocculation reactor (V = 0.55 m^3^) before the particulate matter was removed through a Pile Cloth Media (PCM). The PCMF drum filter unit (Mecana AG) with 2 m^2^ filter surface was operated with Ultrafiber PCM UF-10 and filter velocities were between 1 and 10 m/h. Depending on the v_F_ the HRT infront of the PCMF was 0.5 – 8 min, the volume in the PCMF was between 1.30 m^3^ (minimum water level) and 1.85 m^3^ (switching water level trigger for backwash). The characteristics of the PCM investigated are shown in SI Table 2. The turbidity was measured continuously in the influent and effluent of the pilot plant with two AquaScat 2 WTM (Sigrist Photometer AG).

For the flocculation experiments and turbidity measurements in the laboratory a volume of 20 L was taken as grap samples from the effluent of the PCMF, when no sPAC or FeCl_3_ were added. For the analysis of the residual AC concentration, grab samples as well as 2-h mixed samples with a volume of 20 L from the influent and effluent of the PCMF were taken. To collect the 2-hour mixed sample, a constant flow rate of 0.83 L/min from the PCMF effluent was collected into a sampling bottle. After thorough mixing by shaking, 2 L of the sample was withdrawn from the bottle. This procedure was repeated 10 times over the 2-hour period, resulting in a total of 20 L from the individual 2 L samples. This sampling method also included the backwash cycles of the PCMF (5 – 8 times per hour) into the sampling process. To reach higher turbidity concentrations in the effluent of the PCMF the filter backwash was started manual a few times to create turbidity spikes, during this the grap samples were taken within 2-to-5-minute intervalls.

### 4.5 Analysis of turbidity, absorption_550nm_ and total suspended solids

The turbidity was measured according to (DIN EN ISO 7027-1:2016-11. (2016) with a portable nephelometric turbidimeter (2100Q IS, Hach Lange GmbH) (scattered light measurement). Before each measurement campaign, the turbidity of MilliQ-Water was also measured, which served as the blank value of the instrument and was subtracted from the subsequent turbidity measurements. The blank value was between 0.1 and 0.2 NTU.

Also, measurements of the absorption coefficient at a wavelength of 550 nm (Absorption_550nm_) were determined according to DIN 38404-3:2005-7 (2005) with a quartz glass cell (SUPRASIL®, 50 mm, Hellma Analytics) and a DR 6000 (UV–VIS spectrophotometer, Hach Lange GmbH) with transmitted light measurement. The analysis for TSS was carried out using cellulose nitrate filters (pore size 0.45 µm, Sartorius) with a volume of 1 to 1.5 L of the effluent of the Pile Cloth Media Filter (PCMF) according to DIN-38,409–2 (1987).

### 4.6 Particle size distribution and grey-scale method

The particle size distribution was analysed by dynamic light scattering (DLS) measurements using Mastersizer 3.000 (Malvern Instruments GmbH). The measurements were carried out both with and without the use of sonication (intensity 50%) at 50% at 2000 rpm. For the measurements the automated dispersing unit (Hydro MV) and MilliQ-Water as background matrix were used. The blank values of the MilliQ-Water were automatically subtracted from the measurement results of the particle size analysis by the measuring device via blank value measurements. The measurements were evaluated using Mie theory (Gouesbet and Gréhan 2017). Since the optical material properties of AC match those of carbon black, the absorption index of 1 for carbon black and a refractive index of 1.746 were used (Stagg and Charalampopoulos 1993).

Membrane filters (Cellulose nitrate filterpore size 0.45 µm, Sartorius) were used to create templates for the grey-scale method. Different volumes between 0.1 and 1 L of suspension of MilliQ-Water and the three different AC were filtered through the filters to obtain a filter loading of 0.01 to 1 mg AC/filter. Afterwards the filters were dried at 105 °C for 24 h. To protect the filters and to straighten them, they were laminated and then scanned with an EPSON Perfection V800 scanner (Epson Deutschland GmbH) at 1200 dpi.

### 4.7 Measurement of the residual AC concentration

The analysis of the concentration of AC in the effluent of the PCMF was carried out by Fachhochschule Nordwestschweiz, Institut für Ecopreneurship (Switzerland). For each sample analysis, a triplicate of 500 mL sample volume per replicate was analysed by the mathematical image processing analysis of activated carbon (MIPA^2^C). For quality assurance, a spiking with PAC was carried out for each sample, also in a triplicate. In this case, 0.1 mg PAC/L was added to the 500 mL sample to be analysed. For the MIPA^2^C, the sample was filtered (Whatmann, NC45, 50 mm diameter). Next, the dried filter paper (at 105 °C for at least 4 h) was scanned with an EPSON Perfection V600 scanner at 2400 dpi. The activated carbon concentration was calculated by determining the red, green and blue values used in a multiple polynomial regression model. For three replication the LOD was 0.051 mg/L and the LOQ was 0.102 mg/L.

SI Table 1: Characteristics of the Activated Carbon (AC) used

SI Fig. 1: Particle size distribution of powdered activated carbon with three different particle diameters (PAC = powdered activated carbon, fPAC = fine PAC, sPAC = superfine PAC)

SI Table 2: Characteristics of the OptiFiber® Pile Cloth Media (PCM) Ultrafiber (Fundneider et al. 2023)

SI Table 3: Grey-scale measurements with different amounts of sPAC, fPAC and PAC (Membrane filters, diameter 47 mm, used filter surface for filtration diameter 40 mm, Cellulose nitrate filterpore size 0.45 μm, Sartorius).

## CRediT authorship contribution statement

**Franziska Kirchen:** Writing – review & editing, Visualization, Validation, Resources, Methodology, Investigation, Data curation, Conceptualization. **Thomas Fundneider:** Writing – review & editing, Validation, Data curation, Conceptualization. **Louis Gimmel:** Writing – review & editing, Resources. **Michael Thomann:** Writing – review & editing, Resources. **Michael Pulfer:** Writing – review & editing, Resources. **Susanne Lackner:** Writing – review & editing, Validation, Supervision, Funding acquisition.

## Declaration of competing interest

The authors declare that they have no known competing financial interests or personal relationships that could have appeared to influence the work reported in this paper.

## Data Availability

Data will be made available on request.

## Appendix Supplementary materials

Image, application 1
