# Supplementary material for: Scattered and transmitted light as surrogates for activated carbon residual in advanced wastewater treatment processes: Investigating the influence of particle size
Source: Water Res X. 2024 Apr 9;23:100222. doi: 10.1016/j.wroa.2024.100222 (PMC11031823; doi:10.1016/j.wroa.2024.100222)
Supplement: Supplementary file 1 [file mmc1.pdf]

# Supplementary Information

**Scattered and Transmitted Light as Surrogates for Activated Carbon Residual in Advanced Wastewater Treatment Processes: Investigating the Influence of Particle Size**

Franziska Kirchen[1], Thomas Fundneider[2], Louis Gimmel[1], Michael Thomann[3], Michael Pulfer[3], Susanne Lackner[1*]

[1] Department of Civil and Environmental Engineering Sciences, Institute IWAR, Chair of Water and Environmental Biotechnology, Technical University of Darmstadt, Germany

[2] Mecana AG, Industriestrasse 39, 8864 Reichenburg, Switzerland

[3] Institute for Ecopreneurship, School of Life Sciences FHNW, Hofackerstraße 30, 4132 Muttez, Switzerland

* Corresponding author: s.lackner@iwar.tu-darmstadt.de

SI Table 1: Characteristics of the Activated Carbon (AC) used

| **Characteristics** | | | **AC** |
|---|---|---|---|
| BET surface | | [m²/g] | 946 |
| Iodine adsorption | | [mg/g] | 800 – 1,000 |
| Pore volume | | [cm³/g] | 0.721 |
| | Micro pore volume | [cm³/g] | 0.318 |
| | Meso pore volume | [cm³/g] | 0.348 |
| | Makro pore volume | [cm³/g] | 0.055 |
| Elemental analysis | | | |
| | O | [%] | 1.87 |
| | S | [%] | 0.06 |
| | N | [%] | 0.32 |
| | C | [%] | 78.64 |
| | H | [%] | 1.60 |

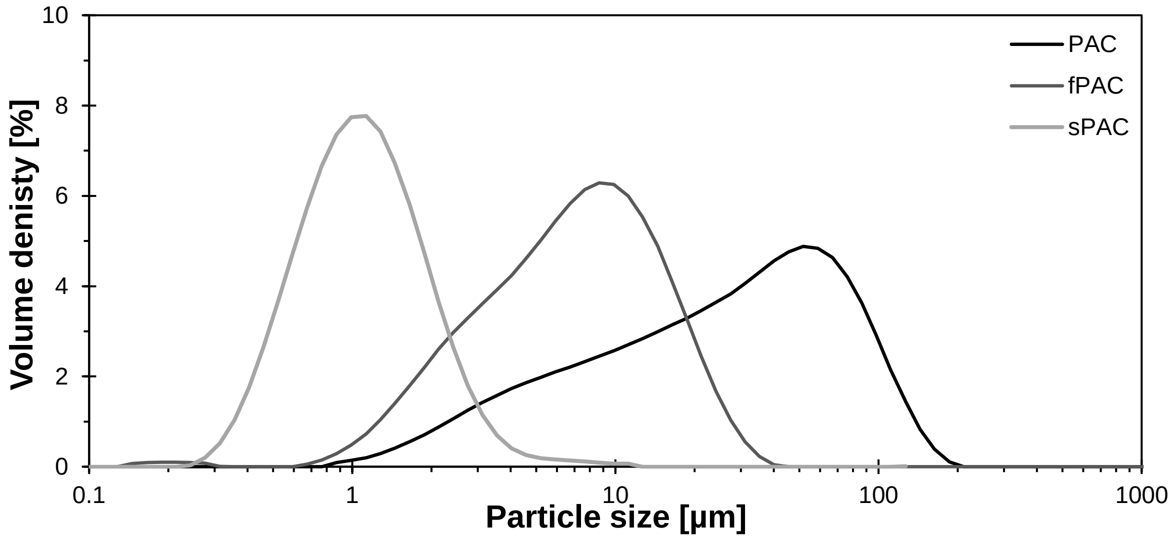


SI Figure 1: Particle size distribution of powdered activated carbon with three different particle diameters (PAC = powdered activated carbon, fPAC = fine PAC, sPAC = superfine PAC)

SI Table 2: Characteristics of the OptiFiber® Pile Cloth Media (PCM) Ultrafiber (Fundneider et al. 2023)

| **Characteristic** | | **OptiFiber® PCM** |
|---|---|---|
| Type | | Ultrafiber |
| Surface weight | [g/m$^2$] | 650 ± 5 % |
| Total thickness | [mm] | 15 ± 1 |
| Air permeability[1] | [l/m$^2$/s] | 434 ± 53 |
| Pile layer | | |
| Material | | PES |
| Fiber surface | [m$^2$/m$^2$] | 260 |
| Thickness | [mm] | 14 ± 1 |
| Filament thickness | [dtex] | 0.26 |
| Filament diameter[2] | [µm] | 4.9 |
| Filament Quantity | [1/m$^2$] | 9.5 x $10^8$ |
| Backing | | |
| Material | | PES and Polypropylene |
| Flow-relevant pores | [µm] | > 400 x 400 |
| Open area | [%] | > 17 |
| Pore quantity | [1/m$^2$] | 1.2 x $10^6$ |
| Filter velocity[3] | [m/h] | 1.0 – 6.5 |

1 DIN EN ISO 9237
2 DIN 65571-1
3 PCMF 2 m$^2$ drum filter unit

SI Table 3: Grey-scale measurements with different amounts of sPAC, fPAC and PAC (Membrane filters, diameter 47 mm, used filter surface for filtration diameter 40 mm, Cellulose nitrate filterpore size 0.45 µm, Sartorius).

| **Mass/Filter** [mg/filter] | **0.05** | **0.2** | **0.4** | **0.6** | **0.8** | **1.0** |
|---|---|---|---|---|---|---|
| **Mass/Filter** [g/m$^2$] | **0.04** | **0.16** | **0.32** | **0.48** | **0.64** | **0.80** |
| **sPAC** | 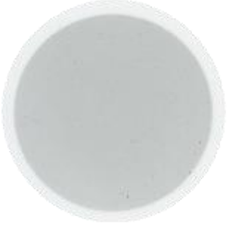 | 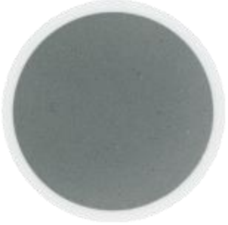 | 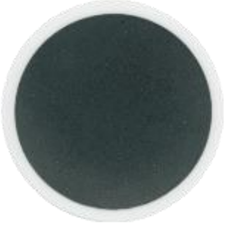 | 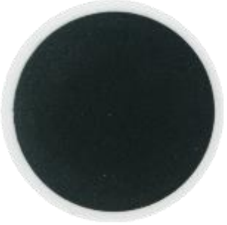 | 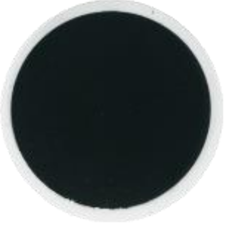 | 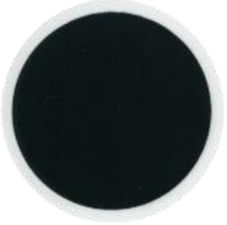 |
| **fPAC** |  | 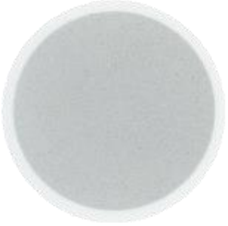 | 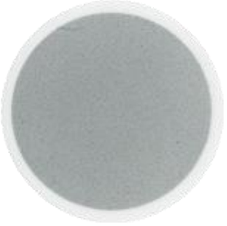 | 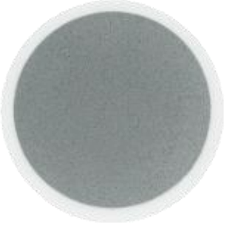 | 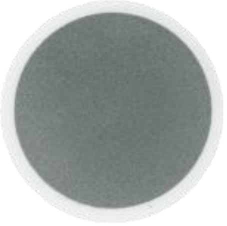 | 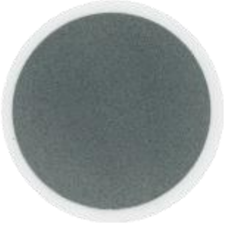 |
| **PAC** | 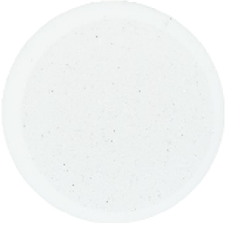 | 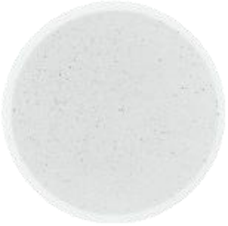 | 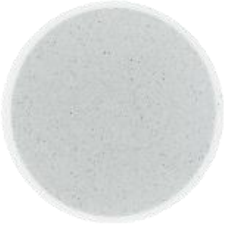 | 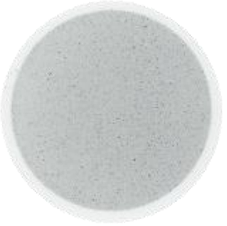 | 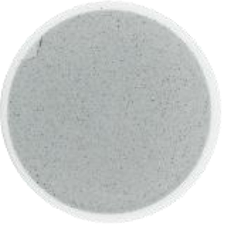 | 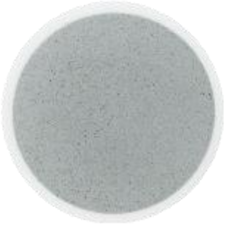 |

## References


DIN 65571-1. 1992. ‘Luft- Und Raumfahrt; Verstärkungsfasern; Bestimmung von Filamentdurchmesser Und Querschnittsfläche von Filamentgarnen; Berechnungsverfahren (Aerospace; Reinforcement Fibres; Determination of Filament Diameter and Cross-Sectional Area of Filament Yarns; Calculation Method)’.

DIN EN ISO 9237. 1995. ‘Textilien - Bestimmung Der Luftdurchlässigkeit von Textilen Flächengebilden (Textiles - Determination of the Air Permeability of Textile Fabrics)’.

Fundneider, Thomas, Franziska Kirchen, Ulrich Grabbe, and Susanne Lackner. 2023. ‘Removal of Superfine Adsorbents with 3D Woven High Performance Optifiber Pile Cloth Media’. In *FILTECH*. Cologne.
